# Effects of a 6-Week Concurrent Training Program Combining Resistance and Various Modalities of Aerobic Exercise in Obese Women with Prehypertension: A Randomized Controlled Trial

**DOI:** 10.3390/metabo15040278

**Published:** 2025-04-17

**Authors:** Jinhyuk Yu, Eunjoo Lee, Jae-Ho Choi, Yerin Sun, Seungyeon Woo, Sohyang Cho, Deunsol Hwang, Sung-Woo Kim, Jisu Kim, Kiwon Lim, Hun-Young Park

**Affiliations:** 1 Department of Sports Medicine and Science, Graduate School, Konkuk University, Seoul 05029, Republic of Korea; jinhyug99@konkuk.ac.kr (J.Y.); eunjooo@konkuk.ac.kr (E.L.); zas1135@konkuk.ac.kr (J.-H.C.); edre82@konkuk.ac.kr (Y.S.); wsyzz92@konkuk.ac.kr (S.W.); whthgiddl123@konkuk.ac.kr (S.C.); hds49@konkuk.ac.kr (D.H.); kswrha@konkuk.ac.kr (S.-W.K.); kimpro@konkuk.ac.kr (J.K.); exercise@konkuk.ac.kr (K.L.); 2 Physical Activity and Performance Institute, Konkuk University, Seoul 05029, Republic of Korea; 3 Department of Physical Education, Konkuk University, Seoul 05029, Republic of Korea

**Keywords:** concurrent training, moderate intensity continuous training, high intensity interval training, prehypertension, obesity, middle-aged women

## Abstract

**Background/Objectives**: Our study aimed to verify the effects of 6 weeks of concurrent training composed of resistance training (RT) and different modalities of aerobic exercise (moderate-intensity continuous training (MICT) or high-intensity interval training (HIIT)) on body composition, blood pressure, vascular function, autonomic nervous system (ANS) function, blood lipid levels, cardiometabolic index (CMI), and health-related fitness in obese middle-aged women with prehypertension. **Methods**: We selected 26 middle-aged women with obesity and prehypertension and divided them equally into the RT + MICT (n = 13) and RT + HIIT (n = 13) groups. The concurrent training program consisted of warm-up, RT, aerobic exercise (MICT or HIIT), and cool-down, and was performed for 6 weeks, three times a week, 85–100 min per session. The measured dependent parameters were analyzed before and after training. **Results**: Concurrent training (RT + MICT and RT + HIIT) for 6 weeks showed significant improvements in body composition, blood pressure, vascular function, ANS function, CMI, and health-related fitness. However, the RT + HIIT group showed a relatively greater improvement in blood lipid levels compared to the RT + MICT group. **Conclusions**: Our study confirmed that both RT + MICT and RT + HIIT yielded similar positive effects on most health-related parameters in obese middle-aged women with prehypertension. Among them, RT + HIIT appeared to be relatively more effective in improving blood lipid profiles.

## 1. Introduction

Obesity refers to a condition that can lead to negative clinical outcomes due to excessive fat accumulation. The Republic of Korea’s Society for the Study of Obesity defines obesity as a body mass index (BMI) of 25 kg/m^2^ or more [1]. According to recent statistical data reported in 2024, the prevalence of obesity in the Republic of Korea has been gradually increasing annually, from 23.4% in 2012 to 27.8% in 2021 [2]. In particular, the prevalence of obesity among women in 2021 was 18.2% in their 20s, 22.4% in their 30s, 26.0% in their 40s, 30.8% in their 50s, and 37.3% in their 60s, showing a rapid increase with age [2].

Various causes of obesity prevalence have been reported, including overeating, lack of sleep, decreased physical activity, irregular lifestyle habits, and westernization of eating habits [3,4].

Comorbidities associated with obesity include heart disease, hypertension, diabetes, cancer, and arthritis [5]. In particular, obesity is associated with the activation of the sympathetic nervous system (SNS) and renin-angiotensin system which, in turn, affect the development of hypertension [6]. The prevalence of hypertension in obese people is approximately three times higher than that in people of normal weight [7], and hypertension caused by obesity is known to increase the risk of cardiovascular disease (CVD) [6]. Hypertension is the most common comorbidity of obesity, causing more than nine million deaths each year [8].

Among the primary modalities for preventing and managing obesity and hypertension, exercise interventions have the advantages of having the fewest side effects and maximizing health benefits by improving insulin resistance, blood glucose, blood pressure, blood lipids, lean body mass, visceral fat, and musculoskeletal function [9,10,11]. Among exercise modalities, concurrent exercise is a combination of resistance and aerobic exercises in a training program that can improve both muscle and cardiopulmonary function [12]. Concurrent exercise can have a positive effect on the prevention and management of obesity and hypertension based on improvements in body fat, abdominal fat, health-related fitness, blood pressure, and vascular function compared to single exercises (e.g., resistance exercise or aerobic exercise) [12]. Therefore, concurrent exercise is considered an effective modality for managing hypertension, obesity, and cardiovascular disease [13].

Today, the key point in creating a concurrent exercise program is the form of aerobic exercise. Aerobic exercise modalities are broadly divided into traditional moderate-intensity continuous training (MICT), and the recently widely used high-intensity interval training (HIIT). MICT is effective at burning fat and improving BMI by supplying sufficient oxygen to active muscles, thereby achieving appropriate aerobic metabolism. This, in turn, positively contributes to increased cardiopulmonary fitness [14,15,16]. HIIT has a positive effect on weight loss, maintenance, and increase in muscle mass, energy expenditure at rest, fat oxidation, and blood glucose control, although it requires a shorter exercise time than MICT to consume the same amount of energy [17,18]. In a previous study comparing the differences in the effects of MICT and HIIT, Mendonça et al. [17] reported that concurrent exercise using MICT or HIIT showed similar effects in reducing body fat, improving aerobic fitness and muscle endurance, and maintaining muscle strength in adolescents; however, concurrent exercise using HIIT was a more time-efficient exercise modality. Dupuit et al. [19] compared the effects of MICT, HIIT, and concurrent exercise using HIIT in postmenopausal women. They reported that all exercise modalities were effective in reducing body weight and body fat mass, but HIIT was more effective in reducing abdominal fat mass than MICT. In particular, adding resistance exercise to HIIT was useful in increasing muscle mass.

Previous studies examining the effects of the concurrent use of MICT and HIIT have various limitations. First, many previous studies that applied concurrent exercise interventions had study periods > 12 weeks [20,21]. Collins et al. [22] reported that 40% of dropouts in studies lasting 12 weeks or longer dropped out because of lack of time, and the dropout rate during the study period was higher in women than in men. In addition, many participants in a 12-week study reported losing interest or dropping out because of the prolonged duration of the intervention [23]. Most previous studies have focused only on fragmentary indicators, such as body composition, blood pressure, and cardiopulmonary endurance [24]. Studies that have simultaneously measured various risk factors that can lead to chronic diseases, such as obesity and hypertension, are limited [25].

Therefore, we examined the effects of a 6-week concurrent training consisting of resistance training (RT) and different modalities of aerobic training (MICT or HIIT) on body composition, blood pressure, vascular function, autonomic nervous system (ANS) function, blood lipid levels, cardiometabolic index (CMI), and health-related fitness in obese middle-aged women with prehypertension.

## 2. Materials and Methods

### 2.1. Participants

This study included 30 middle-aged women who were classified as obese (BMI ≥ 25 kg/m^2^) and had prehypertension (systolic blood pressure (SBP) of 130–139 mmHg or diastolic blood pressure (DBP) of 80–89 mmHg). Participants were randomly assigned in equal numbers to either the RT + MICT group (n = 15), which underwent concurrent training consisting of resistance training and moderate-intensity continuous training (MICT), or the RT + HIIT group (n = 15), which underwent resistance training and high-intensity interval training (HIIT). Participants were excluded if they had a history of severe cerebrovascular or cardiovascular diseases, endocrine or psychiatric disorders, or orthopedic conditions; did not meet the inclusion criteria for body mass index (BMI) or blood pressure; were postmenopausal; smokers; or had a history of alcohol abuse. Individuals who were deemed unsuitable for participation by the investigators were also excluded.

During the study period, four participants withdrew: two from the RT + MICT group (owing to lack of time and work-related issues), and two from the RT + HIIT group (due to family matters and a traffic accident). Consequently, data from 26 participants were included in the final analysis.

The physical characteristics of the participants are presented in Table 1. No significant differences in baseline characteristics were observed between the two groups. All participants received detailed information regarding the purpose and procedures of the study, and provided written informed consent prior to participation. This study was approved by the Institutional Review Board of Konkuk University (KKUIRB-202503-HR-048), Republic of Korea, and was conducted in accordance with the ethical principles of the Declaration of Helsinki.

### 2.2. Study Design

**Figure 1:**
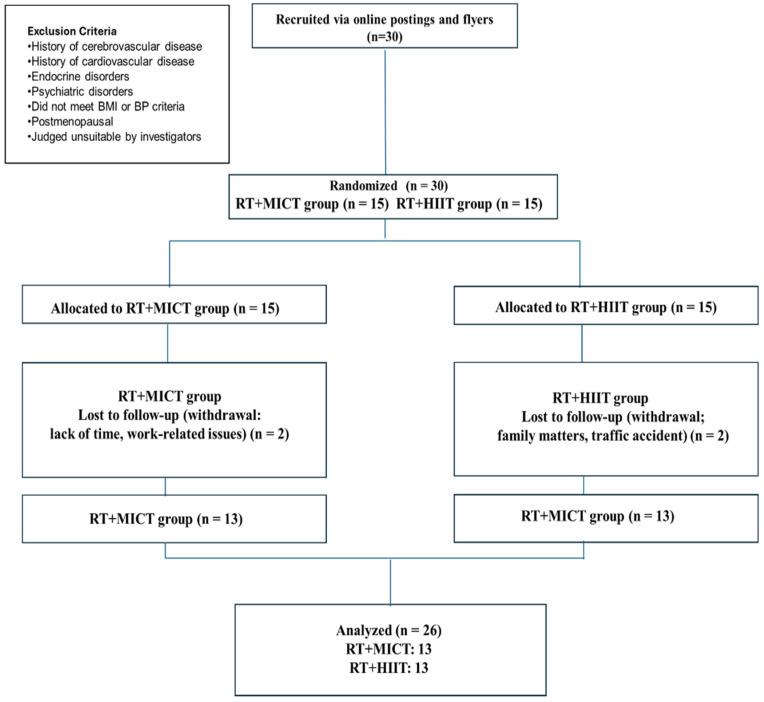
CONSORT flow diagram. MICT, moderate intensity continuous training; HIIT, high intensity interval training; RT, resistance training.

**Figure 2:**
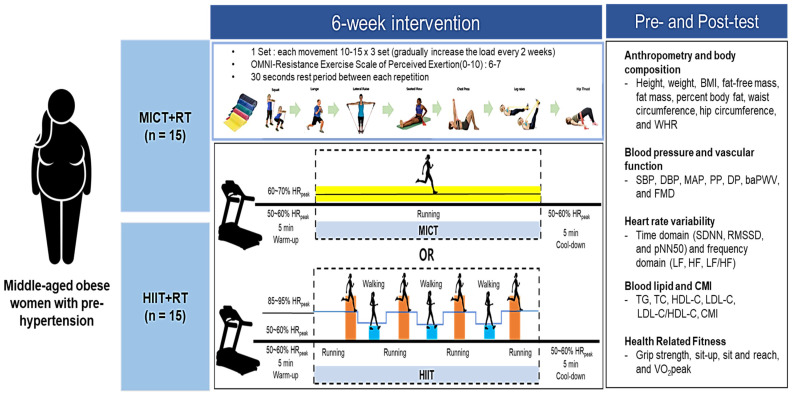
Study design. MICT, moderate intensity continuous training; HIIT, high intensity interval training; RT, resistance training; HRpeak, peak heart rate; BMI, body mass index; WHR, waist and hip ratio; SBP, systolic blood pressure; DBP, diastolic blood pressure; MAP, mean arterial pressure; PP, pulse pressure; DP, double product; baPWV, brachial-ankle pulse wave velocity; FMD, flow mediated dilation; SDNN, standard deviation NN interval; RMSSD, root mean square of the successive differences; pNN50, percentage of NN intervals that differ by more than 50 milliseconds; LF, low frequency; HF, high frequency; TG, triglycerides; TC, total cholesterol; HDL-C, high density lipoprotein-cholesterol; LDL-C, low density lipoprotein-cholesterol; CMI, cardiometabolic index; VO_2_peak, peak oxygen uptake.

The study design is presented in Figure 1, which illustrates the recruitment and allocation of 26 obese middle-aged women with prehypertension. The concurrent training program, consisting of resistance training (RT) combined with different modalities of aerobic exercise (MICT or HIIT), is shown in Figure 2. This program was applied to both groups (RT + MICT and RT + HIIT) and included a warm-up, resistance training, aerobic training (either MICT or HIIT), and a cool-down session. The warm-up consisted of walking or jogging for 10 min at 50–60% of peak heart rate (HRpeak). The RT program lasted for a total of 40 min and included: 5 min of squats, 10 min of lunges, 5 min of lateral raises, 5 min of seated rows, 5 min of chest presses, 5 min of leg raises, and 5 min of hip thrusts. The exercise intensity was set at 6–7 on the OMNI scale, with a progressive overload applied over the six weeks: 10 reps × 3 sets during weeks 1–2, 12 reps × 3 sets during weeks 3–4, and 15 reps × 3 sets during weeks 5–6. A 30-s rest period was provided between exercises. For aerobic exercise, the MICT program was performed for 33 min at 60–70% HRpeak using a treadmill (S25TX, SFET, Seoul, Republic of Korea). The HIIT program consisted of 4 × 4-min high-intensity intervals at 85–95% HRpeak and 3 × 3-min low-intensity intervals at 60–70% HRpeak, for a total of 25 min, using the same treadmill. The cool-down was conducted using the same protocol as the warm-up. The exercise program was designed to ensure equivalent energy expenditure between the MICT and HIIT protocols, based on previous studies [12,21,26]. To ensure participants exercised at the prescribed intensity and maintained proper form, their heart rate was continuously monitored using a heart rate monitor (Polar V800, Polar Electro, Kempele, Finland) throughout each session. The Rating of Perceived Exertion (RPE) was also recorded to assess subjective exercise intensity. All sessions were supervised by certified exercise specialists who provided real-time guidance and feedback to ensure proper execution of the training protocols.

All participants in both groups performed concurrent training for 85–100 min per session, three times a week for 6 weeks, and changes in body composition, blood pressure and vascular function, ANS function, blood lipid level and CMI, and health-related physical fitness before and after training were analyzed in obese middle-aged women with prehypertension. All training and measurements were performed under the same environmental conditions (20 ± 2 °C and 60 ± 2%).

### 2.3. Measurement

Anthropometric and body composition (e.g., height, weight, BMI, lean body mass, body fat percentage, waist circumference (WC), hip circumference (HC), and waist-to-hip ratio (WHR) was measured after fasting for more than 8 h and refrained from strenuous physical activity for 48 h the day before the test using a stadiometer (BSM 330, InBody, Seoul, Republic of Korea), bioelectrical impedance analysis device (Inbody 770; Inbody, Seoul, Republic of Korea), and anthropometric measuring tape (Balzer 80206F, Hoechstmass, Sulzbach, Germany).

Blood pressure was measured using a manual electronic sphygmomanometer (UM-102, A&D Medical, Tokyo, Japan), and pulse, SBP, DBP, mean arterial pressure (MAP), pulse pressure (PP), and myocardial oxygen consumption (double product, DP) were calculated. The following formulas were used: MAP (mmHg) = 1/3 × SBP (mmHg) + 2/3 × DBP (mmHg); PP (mmHg) = SBP (mmHg) − DBP (mmHg); DP (mmHg·bpm) = pulse (bpm) × SBP (mmHg)/100. Arterial stiffness was measured by brachial-ankle pulse wave velocity (baPWV) using VP-1000plus (Omron, Osaka, Japan). The baPWV was calculated by dividing the distance the pressure wave generated in the aorta was transmitted to the peripheral artery during cardiac contraction by the difference in the arrival time. The endothelial function of the arterioles was evaluated by measuring endothelium-dependent vasodilation (flow-mediated dilation, FMD) using noninvasive Doppler ultrasound equipment (UNEXEF38G, Unex, Tokyo, Japan). FMD was calculated using the following formula: FMD (%) = (maximal diameter (mm) − resting diameter (mm)/resting diameter (mm) × 10.

ANS function was evaluated using heart rate variability (HRV), a representative indicator measured using an electrocardiogram (Polar V800, Polat, Kempele, Finland). The measured NN interval data were analyzed using the Kubios HRV (version 3.3.1) program in the time domain for standard deviation of the interval (SDNN), the square root of the mean of the sum of the squares of differences between adjacent NN intervals (RMSSD), and the proportion derived by dividing NN50 by the total number of NN intervals (pNN50), and in the frequency domain for low frequency (LF), high frequency (HF), and LF/HF.

Blood lipid level was measured by collecting 20 uL of blood using the fingertip method with SD LipidoCare (SD Biosensor, Inc., Seoul, Republic of Korea) and injecting it into the Lipid Profile (SD Biosensor Inc., Seoul, Republic of Korea) to measure triglycerides (TG), total cholesterol (TC), low density lipoprotein cholesterol (LDL-C), high density lipoprotein cholesterol (HDL-C), and LDL-C/HDL-C. The CMI was calculated using the following formula, which reflects the risk of metabolic diseases: CMI = [waist circumference (cm)/height (cm)] × [TG (mmol/L)/HDL-C (mmol/L)].

Health-related physical fitness was assessed as follows. Muscle strength was assessed by measuring the grip strength (kg) using a grip dynamometer (T.K.K. 5001; TAKEI, Tokyo, Japan). Muscular endurance was assessed by performing sit-ups for 60 s. Flexibility was assessed by measuring the sit-and-reach distance (cm) by using a sit-and-reach tester (T.K.K. 5403, Flexion-D, Tokyo, Japan). Cardiorespiratory endurance was assessed using grade exercise testing (GXT) to obtain the peak oxygen uptake (VO_2_peak) (mL/kg/min). The GXT was performed using the Bruce protocol with incline and speed increased every 3 min on a treadmill (S25TX, SFET, Seoul, Republic of Korea) while wearing a respiratory gas analyzer (Quark CPET, Cosmed, Roma, Italy) and heart rate monitor (Polar V800, Polar Electro, Kempele, Finland).

### 2.4. Statistical Analysis

Prior to conducting statistical analyses, we determined the required sample size to ensure sufficient statistical power. This study aimed to achieve a statistical power exceeding 90%. A previous study involving obese women reported a partial η^2^ (ηp^2^) of 0.35 [19]. Based on this result, a G*Power analysis was performed by setting the significance level (α) at 0.05, statistical power (1-β) at 0.9, and the effect size (dz) at 0.73. The required sample size to meet these criteria was calculated to be 22 participants. The estimated power based on this sample size exceeded 90%, and the results of the normality test supported the validity of the selected sample size. All statistical analyses were performed using SPSS, version 28.0 (IBM Corp., Armonk, NY, USA). Data are presented as mean ± standard deviation (SD). The normality of the distribution of all outcome variables was verified using the Shapiro–Wilk W-test prior to the parametric tests. To verify the differences between RT + MICT and RT + HIIT concurrent training, two-way analysis of variance (ANOVA) with repeated measures was used to assess the presence of interactions and main effects within the intervention. Partial eta-squared (ηp^2^) values were calculated as measures of effect size for the analysis of variance. When ANOVA revealed a significant interaction or main effect within the intervention, a Bonferroni post hoc test and paired t-test were used to identify within-time differences. To derive clinically meaningful changes due to training in each group, the effect size and 95% confidence intervals (CI) were calculated using Cohen’s d. An effect size of less than 0.2 is defined as very small, 0.2–0.5 as small, 0.5–0.8 as medium, 0.8–1.5 as large, and greater than 1.5 as a very large effect size. The level of significance was set a priori at *p* < 0.05.

## 3. Results

### 3.1. Body Composition

All body composition parameters showed no significant interaction; however, waist circumference (WC) (*p* < 0.001, *η_p_*^2^ = 0.604), hip circumference (HC) (*p* < 0.001, *η_p_*^2^ = 0.753), and waist and hip ratio (WHR) (*p* = 0.030, *η_p_*^2^ = 0.394) showed a main effect within intervention (Table 2). Post hoc analysis indicated that a significant reduction within intervention was observed in WC (RT + MICT: Cohen’s d −1.075, 95% confidence interval [CI] −1.751 to −0.371, *p* = 0.002; RT + HIIT: Cohen’s d −1.313, 95% CI −2.051 to −0.547, *p* < 0.001) and HC (RT + MICT: Cohen’s d −1.579, 95% CI −2.392 to −0.737, *p* < 0.001; RT + HIIT: Cohen’s d −1.272, 95% CI −2.716 to −0.909, *p* < 0.001) in both groups, and RT + MICT and RT + HIIT showed similar effect sizes. Despite a significant main effect within the intervention, WHR did not show significant differences pre- and post-intervention in either group.

### 3.2. Blood Pressure and Vascular Function

Table 3 shows the pre- and post-intervention blood pressure and vascular function data. There was no significant interaction; however, HR (*p* < 0.001, *η_p_*^2^ = 0.482), SBP (*p* < 0.001, *η_p_*^2^ = 0.656), DBP (*p* < 0.001, *η_p_*^2^ = 0.383), MAP (*p* < 0.001, *η_p_*^2^ = 0.543), DP (*p* < 0.001, *η_p_*^2^ = 0.928), baPWV (*p* < 0.001, *η_p_*^2^ = 0.576), and FMD (*p* < 0.001, *η_p_*^2^ = 0.807) showed a main effect within intervention. Post hoc analysis revealed a significant improvement in HR (RT + MICT: Cohen’s d −0.680, 95% CI −1.275 to −0.062, *p* = 0.031; RT + HIIT: Cohen’s d −1.334, 95% CI −2.078 to −0.563, *p* < 0.001), SBP (RT + MICT: Cohen’s d −1.106, 95% CI −1.791 to −0.395, *p* = 0.002; RT + HIIT: Cohen’s d −1.514, 95% CI −2.308 to −0.691, *p* < 0.001), DBP (RT + MICT: Cohen’s d −1.153, 95% CI −1.848 to −0.429, *p* < 0.001; RT + HIIT: Cohen’s d −0.634, 95% CI −1.221 to −0.024, *p* = 0.041), MAP (RT + MICT: Cohen’s d −1.406 95% CI −2.169 to −0.614, *p* < 0.001; RT + HIIT: Cohen’s d −0.928, 95% CI −1.570 to −0.259, *p* = 0.006), DP (RT + MICT: Cohen’s d −3.339, 95% CI −4.756 to −1.904, *p* < 0.001; RT + HIIT: Cohen’s d −3.550, 95% CI −5.044 to −2.038, *p* < 0.001), baPWV (RT + MICT: Cohen’s d −0.921, 95% CI −1.562 to −1.562, *p* = 0.006; RT + HIIT: Cohen’s d −1.326, 95% CI −2.067 to −0.557, *p* < 0.001), and FMD (RT + MICT: Cohen’s d 1.741, 95% CI 0.850 to 2.604, *p* < 0.001; RT + HIIT: Cohen’s d 2.181, 95% CI 1.150 to 3.187, *p* < 0.001) in both groups. Overall, RT + MICT and RT + HIIT showed similar efficacies in terms of blood pressure and vascular function.

### 3.3. Autonomic Nervous System Function

The pre- and post-intervention ANS function data in both groups are shown in Table 4. There was no significant interaction; however, SDNN (*p* = 0.023, *η_p_*^2^ = 0.196), LF (*p* = 0.020, *η_p_*^2^ = 0.207), HF (*p* < 0.001, *η_p_*^2^ = 0.593), and LF/HF (*p* < 0.001, *η_p_*^2^ = 0.447) showed a main effect within intervention. Post hoc analysis indicated that a significant improvement within intervention was observed in HF (RT + MICT: Cohen’s d 0.943, 95% CI 0.270 to 1.589, *p* = 0.005; RT + HIIT: Cohen’s d 1.409, 95% CI 0.617 to 2.174, *p* < 0.001) and LF/HF (RT + MICT: Cohen’s d −0.856, 95% CI −1.484 to −0.203, *p* = 0.009; RT + HIIT: Cohen’s d −0.933, 95% CI −1.577 to −0.263, *p* = 0.006) in both groups. However, LF showed significant improvement only in the MICT + RT group (RT + MICT: Cohen’s d −0.612, 95% CI –1.197 to −0.007, *p* = 0.048). The SDNN showed no differences via the intervention in either group, although a significant main effect was observed within the intervention.

### 3.4. Blood Lipid Level and Cardiometabolic Index

**Figure 3:**
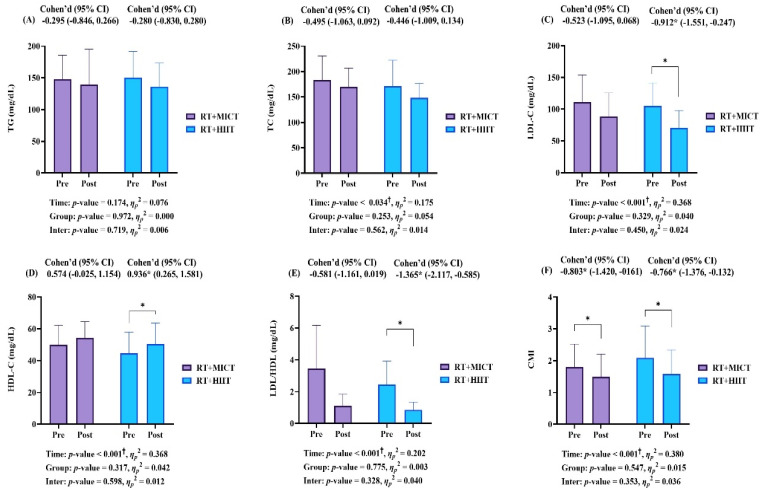
Blood lipid and cardiometabolic index (**A**–**F**). Values are expressed as mean and standard deviation. RT, resistance training; MICT, moderate-intensity continuous training; HIIT, high-intensity interval training; TG, triglycerides; TC, total cholesterol; LDL-C, low density lipoprotein-cholesterol; HDL-C, high density lipoprotein-cholesterol; CMI, cardiometabolic index; CI, confidential interval; G, group; T, time; G × T, interaction. † Significant interaction or main effect. * *p* < 0.05, significant difference between pre- and post-intervention in each group.

Figure 3 shows the pre- and post-intervention blood lipid levels and CMI data. There was no significant interaction; however, TC (*p* = 0.034, *η_p_*^2^ = 0.175), LDL-C (*p* < 0.001, *η_p_*^2^ = 0.368), HDL-C (*p* = 0.001, *η_p_*^2^ = 0.368), LDL-C/HDL-C (*p* < 0.001, *η_p_*^2^ = 0.202), and CMI (*p* < 0.001, *η_p_*^2^ = 0.380) showed a main effect within intervention. As results of post hoc analysis, LDL-C (RT + HIIT: Cohen’s d 0.912, 95% CI −1.551 to −0.247, *p* = 0.006), HDL-C (RT + HIIT: Cohen’s d 0.936, 95% CI 0.265 to 1.581, *p* = 0.006), and LDL-C/HDL-C (RT + HIIT: Cohen’s d −1.365, 95% CI −2.117 to −0.585, *p* < 0.001) showed a significant improvement in only RT + HIIT. However, CMI (RT + MICT: Cohen’s d −0.803, 95% CI −1.420 to −0.161, *p* = 0.013, RT + HIIT: Cohen’s d −0.766, 95% CI −1.376 to −0.132, *p* = 0.017) showed significant improvement in both groups. Accordingly, it was confirmed that RT + HIIT was more effective than RT + MICT in improving blood lipid levels (e.g., LDL-C, HDL-C, and LDL/HDL).

### 3.5. Health-Related Fitness

As shown in Table 5. all health-related fitness parameters showed no significant interaction; however, sit-up (*p* = 0.006, *η_p_*^2^ = 0.275), sit-and-reach (*p* < 0.001, *η_p_*^2^ = 0.555), and VO_2_peak (*p* < 0.001, *η_p_*^2^ = 0.679) showed a main effect within intervention. Post hoc analysis indicated that a significant reduction within the intervention was observed in sit-and-reach (RT + MICT: Cohen’s d 1.640, 95% CI 0.780 to 2.472, *p* < 0.001; RT + HIIT: Cohen’s d 0.732, 95% CI 0.104 to 1.336, *p* = 0.022) and VO_2_peak (RT + MICT: Cohen’s d 1.100, 95% CI 0.390 to 1.783, *p* = 0.002; RT + HIIT: Cohen’s d 1.882, 95% CI 0.947 to 2.789, *p* < 0.001). The sit-up showed no differences via the intervention in either group, although a significant main effect within the intervention was observed.

## 4. Discussion

We hypothesized that 6 weeks of concurrent training using moderate-intensity continuous training (MICT) or high-intensity interval training (HIIT) combined with resistance training (RT) will have a positive effect on body composition, blood pressure, vascular function, ANS function, blood lipid concentration, and health-related physical fitness in obese middle-aged women with prehypertension, and RT + HIIT will be more effective than RT + MICT. Consistent with this hypothesis, RT + HIIT was relatively more effective than RT + MICT in improving blood lipid levels (e.g., LDL-C, HDL-C, LDL/HDL); however, the other parameters showed a similar positive effect with the 6 weeks of concurrent training in both groups.

Previous studies have extensively investigated the effects of MICT and HIIT with or without RT on body composition and reported conflicting results depending on the characteristics of the participants, exercise duration, and exercise intensity [26,27]. Sun et al. [26] assessed and compared the effect of an 8-week HIIT or MICT program on the body composition outcomes of sedentary adolescents in China and reported both 8-week HIIT and MICT programs have similar positive effects on reducing body fat mass, fat percentage, and visceral fat area. Sanca-Valeriano et al. [28] and Wewege et al. [27] conducted systematic reviews and meta-analyses comparing the effects of HIIT and MICT in overweight and obese adults. They reported that in intervention programs averaging 6–16 weeks with three sessions per week, there were no significant differences between the two exercise modalities across various body composition measures, including weight, BMI, waist circumference, and body fat mass. In contrast, Dupuit et al. [19] investigated the effects of MICT, HIIT, and HIIT + RT programs (three sessions per week for 12 weeks) on the body composition of overweight and obese postmenopausal women. This study found that all programs contributed to weight loss and a reduction in total fat mass (FM). However, HIIT was more effective than MICT in reducing abdominal and visceral fat, while the addition of resistance training (RT) did not further enhance this effect but had a positive impact on increasing muscle mass percentage. These findings suggest that concurrent training promotes both muscle gain and fat reduction, indicating that body composition can be improved without significant changes in body weight. Additionally, since the duration of this study was six weeks, a relatively short period that falls within the exercise adaptation phase, there is a possibility that increases in body water and muscle mass may have offset the effects of weight loss [29,30].

In the present study, we confirmed that 6 weeks of all concurrent training (RT + MICT and RT + HIIT) significantly improved WC and HC in obese middle-aged women with prehypertension. This suggests that HIIT can be a time-efficient exercise modality for body composition, as it showed similar effects despite the shorter exercise time of RT + HIIT than that of RT + MICT [27]. However, there were no significant improvements in body weight, BMI, lean body mass, fat mass, or body fat percentage. These results are probably due to the relatively short concurrent training period of six weeks. In addition, because the present study used a combination of resistance exercise and two types of aerobic exercise (RT + MICT and RT + HIIT), we expect that a longer period of exercise intervention will have a clearer positive effect on body composition parameters [19].

Regarding blood pressure and vascular function, a number of studies have compared the effects of MICT and HIIT intervention combined with or without RT in various populations [31,32]. Taylor et al. [33] compared the effects of 4-week HIIT and MICT on systemic vascular function and stiffness in patients with coronary artery disease undergoing a cardiac rehabilitation program and concluded that a 4-week HIIT program was superior to MICT for improving vascular function, but not arterial stiffness or blood pressure. Shishira et al. [32] investigated the effects of HIIT versus MICT on vascular function, including arterial diameter, arterial stiffness, pulse wave velocity, and blood flow in overweight and obese individuals. They concluded that HIIT is a more effective and time-efficient modality for improving vascular function in overweight and obese individuals, leading to enhancements in FMD by 3.9% and arterial diameter by 4.8% compared to MICT. HIIT is a high-intensity exercise involving repeated exposure to strong blood flow stimuli. Consequently, NO production in endothelial cells increases rapidly, promoting vasodilation, which makes it more effective in improving FMD [34,35]. Li et al. [29] performed a meta-analysis to compare the effects of HIIT and MICT on blood pressure in patients with essential hypertension and explored more suitable intervention modalities. They reported that HIIT and MICT had similar effects on the overall resting SBP and DBP in patients with hypertension and prehypertension; however, HIIT was better than MICT in reducing SBP and improving vasodilation. Sabouri et al. [30] conducted a meta-analysis including eight studies with 208 participants, quantified the effect of HIIT on FMD in overweight/obese adults, and reported that HIIT improved vascular endothelial function with an overall change of 2.6% and 1.83% compared to MICT and control, respectively, in overweight and obese adults. As mentioned in previous studies, both MICT and HIIT improve blood pressure and vascular function; however, HIIT has been reported to be more effective than MICT. In contrast, Du et al. [36] explored the effects of MICT and HIIT on key risk factors associated with metabolic syndrome (MetS), and concluded that MICT appears to offer a specific benefit in reducing SBP, making it more suitable for patients with MetS with elevated SBP, whereas HIIT might be preferable for individuals with limited time or difficulty adhering to regular exercise. In the present study, we confirmed that RT + MICT and RT + HIIT showed positive improvements in resting HR, SBP, DBP, MAP, DP, baPWV, and FMD; however, both interventions showed similar efficacy in blood pressure and vascular function in obese middle-aged women with prehypertension. These results are likely due to the exercise volume being sufficient by performing RT in addition to aerobic exercise corresponding to MICT or HIIT, despite the relatively short intervention period of 6 weeks, thus resulting in positive effects on blood pressure and vascular function in both groups. In the present study, both RT + HIIT and RT + MICT led to positive improvements in vascular function indicators such as blood pressure, baPWV, and FMD; however, no clear difference was observed between the two groups. This outcome can be attributed to several factors. First, previous studies have shown that the vascular benefits of HIIT typically become more pronounced after relatively long intervention periods, generally exceeding 8 weeks, whereas the present study was limited to a 6-week period [32]. Second, concurrent training, which included resistance training, might have independently contributed to improvements in blood flow and endothelial function, potentially offsetting the differences between aerobic exercise modalities (HIIT vs. MICT) [37]. Third, the participants in this study were middle-aged obese women with prehypertension, a population that may have limited endothelial responsiveness or vascular reactivity to high-intensity stimuli [38].

HRV evaluation reflects the activity and balance of the SNS and parasympathetic nervous system (PNS), which modulate cardiovascular function and are used to evaluate ANS function [39]. HRV is generally used to verify fluctuations in ANS activity and balance due to stress and to obtain information on stress-related diseases. Regarding HRV parameters, LF is highly correlated with mental stress caused by SNS activation, indicating cardiac instability [40]. The HF mainly reflects the activity of the vagus nerve branching to the heart and is a representative parameter of PNS activity, indicating a high level of ANS function [41]. Additionally, LF/HF reflects the overall balance of the ANS, with higher values indicating that the SNS is relatively more active or that the PNS is more inactive [39]. Philbois et al. [42] investigated whether the benefits of HIIT related to cardiovascular autonomic control were greater than those of MICT in women with polycystic ovarian syndrome, and reported that 16 weeks of HIIT and MICT showed similar results for ANS function. Su et al. [43] hypothesized that HIIT may be more effective than MICT in improving ANS balance in obese adolescent boys, and confirmed that 8 weeks of HIIT may be superior to MICT in improving ANS balance assessed using HRV. Alansare et al. [44] compared the effects of 2 weeks of HIIT versus MICT on HRV in physically inactive adults as a preliminary study, and they concluded that HIIT is superior to MICT in improving HRV and can be applied as a time-efficient program for improving cardiac-autoregulation in physically inactive adults. In the present study, we confirmed that both RT + MICT and RT + HIIT showed positive improvements in ANS function (e.g., HF and LF/HR), despite the short intervention period, unlike the study by Philbois et al. [42]. Considering that previous studies with relatively short intervention periods have reported that HIIT is more effective than MICT in enhancing ANS function [43,44], the absence of a clear difference between the two groups in the present study may be attributed to the addition of resistance training to both aerobic exercise modalities (MICT and HIIT), which likely provided sufficient stimulation to the neuromuscular and autonomic nervous systems [45]. Furthermore, the concurrent training approach may have ensured adequate exercise volume over the relatively short 6-week period. It is also possible that the inclusion of resistance training itself offered enough autonomic stimulation to offset the differences between HIIT and MICT [45,46]. Lastly, since the participants in this study were middle-aged obese women who tend to have lower autonomic regulation and reduced recovery capacity, a clear autonomic response to high-intensity exercise such as HIIT may have been difficult to elicit [46].

Regarding blood lipid and CMI, both MICT and HIIT combined with RT can improve blood lipid level and cardiometabolic health; however, the effects of each intervention modality vary depending on the study and the specific metrics being measured [26,47,48]. Da Silva et al. [47], with the most similar design to the present study, analyzed the effects of different aerobic exercise modes and intensities (i.e., HIIT vs. MICT combined with an RT program) on metabolic outcomes in participants with MetS, and suggested that both concurrent trainings for 12 weeks promote important cardiometabolic gains, particularly in the RT + HIIT group. McCormick et al. [48] performed a meta-analysis to compare the effects of HIIT versus MICT on blood lipid levels in non-diabetic overweight and obese young adults and reported that HIIT is superior to MICT in improving LDL and TC. In the present study, CMI showed significant improvement in both groups; however, LDL-C, HDL-C, and LDL-C/HDL-C showed significant improvement only in the RT + HIIT. These results are consistent with those of the previous studies mentioned above, and confirmed that RT + HIIT was relatively more effective than RT + MICT in improving blood lipid levels.

Both MICT and HIIT with or without RT can improve health-related fitness, such as muscle strength, endurance, cardiorespiratory fitness, and exercise performance [17,49,50]. Mendonça et al. [17] compared the effects of two concurrent training modalities on health-related physical fitness among adolescents. They suggested that 12 weeks of training using MCIT + RT or HIIT + RT showed a similar effect on health-related physical fitness components, such as muscular and cardiopulmonary fitness, in adolescents. Guo et al. [50] evaluated the effect of HIIT and MICT on cardiorespiratory fitness in young and middle-aged individuals via a systematic review and meta-analysis, and concluded that the effect of HIIT on cardiorespiratory fitness in young and middle-aged individuals was similar to or better than that of MICT, which might be influenced by age (18–45 years), complications (obesity), duration (>6 weeks), frequency, and HIIT interval. Yakut et al. [51] compared the effects of home-based HIIT and MICT in patients with myocardial infarction, and suggested that HIIT and MICT can be practiced at home in patients with MI and play an important role in improving functional capacity and health outcomes. In the present study, we confirmed that both RT + MICT and RT + HIIT showed positive improvements in flexibility (e.g., sit-and-reach) and cardiopulmonary fitness (e.g., VO_2_peak), as in previous studies. Considering previous studies showing that concurrent training composed of RT and aerobic training improve health-related physical fitness in various populations [52,53,54], it seems desirable that flexibility and cardiopulmonary fitness are improved in both RT + MICT and RT + HIIT. Recent research suggests that exercise timing may influence the effectiveness of training on cardiometabolic health. For instance, Morales-Palomo et al. [54] reported that afternoon aerobic exercise resulted in greater improvements in metabolic syndrome components than morning sessions. Based on these findings, future studies should consider exercise timing as a potentially important factor when designing interventions.

Although this study yields meaningful results, it has several limitations. First, all participants were middle-aged obese women with prehypertension. However, their menstrual cycle phases and sex hormone levels were not assessed, which could have influenced their cardiometabolic and autonomic nervous system responses. Second, physical activity levels and dietary intake were not monitored during the intervention, potentially acting as confounding factors. Third, the 6-week intervention period was relatively short and chosen to enhance adherence, limiting direct comparisons with studies using longer durations (e.g., ≥8 weeks). Fourth, although the normality of the data was confirmed using the Shapiro–Wilk W-test, the small sample size may have limited statistical power and generalizability. Finally, recent evidence suggests that exercise timing can affect cardiometabolic outcomes; however, our study did not control for or analyze exercise timing, which might have influenced the results.

In conclusion, both RT + MICT and RT + HIIT led to similar improvements in body composition, vascular function, autonomic nervous system function, and health-related physical fitness. Notably, RT + HIIT was more effective than RT + MICT in improving blood lipid profiles. These findings suggest that both types of concurrent training can effectively improve health outcomes in obese middle-aged women with prehypertension. Future studies should consider longer intervention periods, larger sample sizes, and better control for confounding factors to further clarify the differential effects of each exercise modality.

## 5. Limitations

Our study had several limitations. First, participants in our study were middle-aged obese women with prehypertension; however, their menstrual cycles and sex hormone levels were not evaluated. Second, our study did not investigate the amount of physical activity or dietary intake of the participants during the intervention period. Third, because the present study utilized a relatively short intervention period of 6 weeks to increase the exercise compliance of the participants, it is difficult to generalize the results of our study to those of previous studies with different intervention periods (i.e., 2 to 16 weeks). Fourth, the normality of the distribution of all outcome parameters was verified using the Shapiro–Wilk W-test prior to the parametric tests; however, the small sample size may limit the interpretation of the results of this study. Lastly, although previous studies have reported that the timing of exercise may influence its effectiveness, particularly on cardiometabolic health markers, our study did not control for or analyze the potential effects of exercise timing, which might have influenced the results.

## 6. Conclusions

Our study demonstrated that both the RT + MICT and RT + HIIT programs, when performed over 6 weeks, had similar positive effects on most parameters (e.g., body composition, blood pressure and vascular function, ANS function, blood lipid and CMI, and health-related fitness) in obese middle-aged women with prehypertension. However, the 6-week RT + HIIT program was found to be a more time-efficient exercise modality that, despite its relatively shorter duration compared with RT + MICT, was relatively more effective in improving blood lipid levels.

## Figures and Tables

**Table 1:** Participant characteristics.

Parameters	RT + MICT (n = 13)	RT + HIIT (n = 13)	*p*-Value
Age (year)	47.08 ± 6.36	47.00 ± 7.58	0.978
Height (cm)	159.60 ± 3.01	160.75 ± 3.81	0.421
Weight (kg)	70.66 ± 8.77	70.54 ± 9.76	0.973
BMI (kg/m^2^)	27.68 ± 3.86	27.34 ± 3.30	0.808
Lean body mass (kg)	43.72 ± 2.85	43.42 ± 4.71	0.842
Fat mass (kg)	26.82 ± 7.49	27.25 ± 5.52	0.869
Percent body fat (%)	37.42 ± 5.09	38.30 ± 4.10	0.633
Body fat mass (kg)	127.00 ± 6.12	128.15 ± 7.79	0.678
Percent body fat (%)	81.62 ± 6.78	80.15 ± 5.93	0.564

Note. RT, resistance training; MICT, moderate-intensity continuous training; HIIT, high-intensity interval training; BMI, body mass index.

**Table 2:** Body composition.

Parameters	Groups	Test		Cohen’s d (95% CI)	*p* (*η*^2^)	
		Pre	Post			
Body weight (kg)	RT + MICT	70.66 ± 8.77	69.80 ± 9.00	−0.336 (−0.889, 0.230)	T G T × G	0.770 (0.124) 0.944 (0.000) 0.820 (0.002)
	RT + HIIT	70.54 ± 9.76	69.43 ± 8.48	−0.434 (−0.996. 0.143)		
BMI (kg/m^2^)	RT + MICT	27.68 ± 3.86	27.23 ± 3.12	−0.339 (−0.892, 0.227)	T G T × G	0.770 (0.124) 0.831 (0.002) 0.790 (0.003)
	RT + HIIT	27.34 ± 3.30	27.00 ± 3.45	−0.435 (−0.998, 0.143)		
Lean body mass (kg)	RT + MICT	43.72 ± 2.85	43.80 ± 2.61	0.035 (−0.509, 0.578)	T G T × G	0.620 (0.010) 0.889 (0.001) 0.772 (0.004)
	RT + HIIT	43.42 ± 4.71	43.71 ± 4.27	0.197 (−0.356, 0.742)		
Fat mass (kg)	RT + MICT	26.82 ± 7.49	26.14 ± 6.51	−0.254 (−0.802, 0.304)	T G T × G	0.175 (0.075) 0.868 (0.001) 0.988 (0.000)
	RT + HIIT	27.25 ± 5.52	26.55 ± 6.44	−0.299 (−0.850, 0.263)		
Percent body fat (%)	RT + MICT	37.42 ± 5.09	37.18 ± 4.78	−0.127 (−1.751, −0.371)	T G T × G	0.218 (0.063) 0.757 (0.004) 0.491 (0.020)
	RT + HIIT	38.30 ± 4.10	37.45 ± 5.51	−0.346 (−0.900, 0.221)		
WC (cm)	RT + MICT	89.82 ± 6.68	87.95 ± 6.32	−1.075 * (−1.751, −0.371)	T G T × G	<0.001 ^†^ (0.604) 0.544 (0.016) 0.787 (0.003)
	RT + HIIT	88.02 ± 8.92	85.98 ± 9.05	−1.313 * (−2.051, −0.547)		
HC (cm)	RT + MICT	104.35 ± 6.61	101.65 ± 6.90	−1.579 * (−2.392, −0.737)	T G T × G	<0.001 ^†^ (0.753) 0.889 (0.001) 0.754 (0.004)
	RT + HIIT	103.88 ± 6.80	101.38 ± 6.93	−1.272 * (−2.716, −0.909)		
WHR	RT + MICT	0.86 ± 0.05	0.87 ± 0.05	0.256 (−0.302, 0.804)	T G T × G	0.030 ^†^ (0.394) 0.889 (0.001) 0.555 (0.015)
	RT + HIIT	0.85 ± 0.06	0.85 ± 0.07	0.061 (−0.484, 0.604)		

Note. Values are expressed as mean and standard deviation. RT, resistance training; MICT, moderate-intensity continuous training; HIIT, high-intensity interval training; BMI, body mass index; WC, waist circumference; HC, hip circumference; WHR, waist-to-hip ratio; CI, confidence interval; G, group; T, time; G × T, interaction. † Significant interaction or main effect. * *p* < 0.05, significant difference between pre- and post-intervention in each group.

**Table 3:** Blood pressure and vascular function.

Parameters	Groups	Test		Cohen’s d (95% CI)	*p* (*η*^2^)	
		Pre	Post			
HR (bpm)	RT + MICT	72.20 ± 8.99	66.75 ± 11.59	−0.680 * (−1.275, −0.062)	T G T × G	<0.001 ^†^ (0.482) 0.727 (0.005) 0.514 (0.018)
	RT + HIIT	71.91 ± 7.10	64.68 ± 8.47	−1.334 * (−2.078, −0.563)		
SBP (mmHg)	RT + MICT	127.00 ± 6.12	123.77 ± 6.94	−1.106 * (−1.791, −0.395)	T G T × G	<0.001 ^†^ (0.656) 0.968 (0.000) 0.069 (0.132)
	RT + HIIT	128.15 ± 7.79	122.38 ± 8.45	−1.514 * (−2.308, −0.024)		
DBP (mmHg)	RT + MICT	81.62 ± 6.78	77.85 ± 5.46	−1.153 * (−1.848, 0.429)	T G T × G	<0.001 ^†^ (0.383) 0.416 (0.028) 0.799 (0.003)
	RT + HIIT	80.15 ± 5.93	75.85 ± 5.58	−0.634 * (−1.221, −0.024)		
MAP (mmHg)	RT + MICT	96.77 ± 6.13	93.08 ± 5.24	−1.406 * (−2.169, −0.614)	T G T × G	<0.001 ^†^ (0.543) 0.559 (0.014) 0.588 (0.012)
	RT + HIIT	96.00 ± 5.15	91.46 ± 5.39	−0.928 * (−1.570, −0.259)		
PP (mmHg)	RT + MICT	45.38 ± 4.81	45.92 ± 6.33	0.143 (−0406, 0.687)	T G T × G	0.636 (0.009) 0.522 (0.017) 0.348 (0.037)
	RT + HIIT	48.15 ± 8.75	46.54 ± 8.31	−0.225 (−0.771, 0.330)		
DP (mmHg ·bpm)	RT + MICT	8932.23 ± 1169.78	5425.85 ± 941.12	−3.339 * (−4.756, −1.904)	T G T × G	<0.001 ^†^ (0.928) 0.551 (0.015) 0.824 (0.002)
	RT + HIIT	8783.85 ± 891.30	5186.69 ± 827.34	−3.550 * (−5.044, −2.038)		
baPWV (cm/s)	RT + MICT	1316.92 ± 128.52	1254.69 ± 163.17	−0.921 * (−1.562, −1.562)	T G T × G	<0.001 ^†^ (0.576) 0.952 (0.000) 0.371 (0.034)
	RT + HIIT	1325.08 ± 170.14	1239.15 ± 166.56	−1.326 * (−2.067, −0.557)		
FMD (%)	RT + MICT	5.14 ± 0.98	5.73 ± 0.69	1.741 * (0.850, 2.604)	T G T × G	<0.001 ^†^ (0.807) 0.473 (0.022) 0.189 (0.071)
	RT + HIIT	5.30 ± 1.08	6.08 ± 0.82	2.181 * (1.150, 3.187)		

Note. Values are expressed as mean and standard deviation. RT, resistance training; MICT, moderate-intensity continuous training; HIIT, high-intensity interval training; HR, heart rate; SBP, systolic blood pressure; DBP, diastolic blood pressure; MAP, mean arterial pressure; PP, pulse pressure; DP, double product; baPWV, brachial-ankle pulse wave velocity; FMD, flow-mediated dilation; CI, confidence interval; G, group; T, time; G × T, interaction. † Significant interaction or main effect. * *p* < 0.05, significant difference between pre- and post-intervention in each group.

**Table 4:** Autonomic nervous system function.

Parameters	Groups	Test		Cohen’s d (95% CI)	*p* (*η*^2^)	
		Pre	Post			
SDNN (ms)	RT + MICT	24.32 ± 11.04	30.91 ± 16.23	0.433 (−0.145, 0.995)	T G T × G	0.023 ^†^ (0.196) 0.986 (0.000) 0.846 (0.002)
	RT + HIIT	24.75 ± 7.89	30.35 ± 10.10	0.565 (−0.032, 1.143)		
RMSSD (ms)	RT + MICT	26.67 ± 20.51	31.18 ± 14.90	0.225 (−0.331, 0.771)	T G T × G	0.222 (0.061) 0.950 (0.000) 0.819 (0.002)
	RT + HIIT	27.70 ± 9.52	30.80 ± 13.26	0.356 (−0.213, 0.910)		
pNN50 (%)	RT + MICT	8.83 ± 13.56	10.66 ± 16.90	0.188 (−0.365, 0.733)	T G T × G	0.155 (0.082) 0.741 (0.005) 0.632 (0.010)
	RT + HIIT	9.61 ± 7.50	13.24 ± 14.94	0.393 (−0.180, 0.951)		
LF (ms^2^)	RT + MICT	394.77 ± 280.91	253.34 ± 105.96	−0.612 * (−1.197, −0.007)	T G T × G	0.020 ^†^ (0.207) 0.986 (0.000) 0.954 (0.000)
	RT + HIIT	397.03 ± 301.33	248.81 ± 138.52	−0.426 (−0.987, 0.151)		
HF (ms^2^)	RT + MICT	124.70 ± 49.96	284.27 ± 153.35	0.943 * (0.270, 1.589)	T G T × G	<0.001 ^†^ (0.593) 0.120 (0.098) 0.427 (0.027)
	RT + HIIT	161.81 ± 75.50	371.97 ± 180.98	1.409 * (0.617, 2.174)		
LF/HF (%)	RT + MICT	3.45 ± 2.72	1.12 ± 0.73	−0.856 * (−1,484, −0.203)	T G T × G	<0.001 ^†^ (0.447) 0.168 (0.078) 0.421 (0.027)
	RT + HIIT	2.45 ± 1.46	0.85 ± 0.49	−0.933 * (−1.577, −0.263)		

Note. Values are expressed as mean and standard deviation. RT, resistance training; MICT: moderate-intensity continuous training; HIIT: high-intensity interval training; SDNN, standard deviation NN interval; RMSSD, root mean square of successive differences; pNN50, percentage of NN intervals that differed by more than 50 ms; LF, low frequency; HF, high frequency; CI, confidence interval; G: group, T, time; G × T, interaction. † Significant interaction or main effect. * *p* < 0.05, significant difference between pre- and post-intervention in each group.

**Table 5:** Health-related fitness.

Parameters	Groups	Test		Cohen’s d (95% CI)	*p* (*η*^2^)	
		Pre	Post			
Grip strength (kg)	RT + MICT	24.62 ± 4.31	24.95 ± 4.10	0.230 (−0.326, 0.776)	T G T × G	0.150 (0.084) 0.644 (0.009) 0.579 (0.013)
	RT + HIIT	25.26 ± 4.89	26.00 ± 5.48	0.342 (−0.225, 0.896)		
Sit-up (n)	RT + MICT	11.31 ± 6.87	13.23 ± 7.84	0.627 (0.019, 1.214)	T G T × G	0.006 ^†^ (0.275) 0.635 (0.010) 0.901 (0.001)
	RT + HIIT	12.77 ± 7.37	14.54 ± 7.88	0.559 (−0.038, 1.136)		
Sit and reach (cm)	RT + MICT	10.24 ± 6.33	12.83 ± 6.95	1.640 * (0.780, 2.472)	T G T × G	<0.001 ^†^ (0.555) 0.785 (0.003) 0.291 (0.046)
	RT + HIIT	10.02 ± 5.17	11.75 ± 5.79	0.732 * (0.104, 1.336)		
VO_2_peak (mL/kg/min)	RT + MICT	31.84 ± 3.39	39.73 ± 5.84	1.100 * (0.390, 1.783)	T G T × G	0.001 ^†^ (0.679) 0.574 (0.013) 0.546 (0.015)
	RT + HIIT	31.78 ± 3.28	41.16 ± 4.40	1.882 * (0.947, 2.789)		

Note. Values are expressed as mean and standard deviation. RT, resistance training; MICT, moderate-intensity continuous training; HIIT, high-intensity interval training; VO_2_peak, peak oxygen uptake; CI, confidence interval; G, group; T, time; G × T, interaction. † Significant interaction or main effect. * *p* < 0.05, significant difference between pre- and post-intervention in each group.

## Data Availability

The data presented in this study are available upon request from the corresponding author.
